# Medical and Surgical Honours : A Suggestion for Reform

**Published:** 1910-02-26

**Authors:** 


### Editor's Letter-Box.

### MEDICAL AND SURGICAL HONOURS: A SUGGESTION FOR REFORM.

*To the Editor of* The Hospital.

Sir,—For many years much irritation has been felt and expressed by medical men about the curious and various estimation in which degrees and diplomas are held in this country. There are many men of great talent, energy, and probity, who through no fault of their own—most of them through lack of means or particular place of residence in youth—are not able reasonably to arrive at a University degree or a College Fellowship—one or other of these two being now practically a *sine qua non* for any public hospital appointment. Such men are thus at the outset of their careers very unfairly handicapped in the race for place and public honour in the profession.

It is not reasonable that such a bar should be, as it now almost always is, practically a perpetual check to a normal ambition. Moreover, in spite of the supervision of the General Medical Council, we are somewhat disposed to believe that the standards exacted by the various examining bodies are of varying value, and that most of the newer schools have scarcely any reputation as yet to boast of.

The suggestion I have to offer, if adopted, would put the young schools upon their mettle and give them a chance of proving their status exactly. Even if we assume that the preliminary education of a graduate is superior to that of a licentiate, and that the differences of talent and usefulness are roughly well recognised by the differences in professional standing and public reputation, should this preliminary mistake constitute a persistent life-long and fatal bar to one who was not so fortunate as to have his career properly mapped out for him in his youth? Some remedy, it seems to me, is possible, and should be brought about quickly.

The remedy I suggest is (1) That none of the Universities should be allowed to grant any degree beyond that of Bachelor in Medicine or Surgery; that none of the Colleges grant more than a Licentiate or Membership. These would include, as now, higher qualifications. (2) That all Bachelors, Licentiates, or Members of so many, say, eight or ten years' standing might then be admitted to one or two examinations conducted by representatives of all the Universities or Colleges, and that that central body may grant degrees or higher qualifications, say M.D., F.R.C.S., Gt. Brit., or to preserve nationality, M.D., F.R.C.S.Eng. Ireland or Scotland as the case may be.

At the present time the best degrees are the M.D.Durh., granted after 40 years of age to men after 15 years' practice, and the F.R.C.S. of the Colleges to which men of mature years and only real practical surgical skill are admitted.

Each University and College under this improved style would exert itself to fit its men for the final coping-stone examination and take rank accordingly. One portal would be open to all Members, Graduates, and Licentiates alike—it would matter little where preliminary education was got—the final examination. A better educated or more worthy man than a Licentiate or Member of any of the Colleges it would be hard to find at present, yet an M.B., easily becoming an M.D. in a year or two, takes social and professional standing quite out of all proportion to his actual merits.

Under this scheme I have outlined, every University and College would vie with one another to try and send most men up to the final and open and best test of real worth. As national distinctions are very much in the air at present, it might be better to favour three medical and surgical portals instead of one—namely, one for England, one for Scotland, and one for Ireland.

Yours truly,
A General Practitioner.

